# Dosimetric accuracy and clinical quality of Acuros XB and AAA dose calculation algorithm for stereotactic and conventional lung volumetric modulated arc therapy plans

**DOI:** 10.1186/1748-717X-8-149

**Published:** 2013-06-24

**Authors:** Petra S Kroon, Sandra Hol, Marion Essers

**Affiliations:** 1 Department of Medical Physics, Institute Verbeeten, Brugstraat 10, 5042 SB Tilburg, the Netherlands; 2 Department of Radiotherapy, Institute Verbeeten, Brugstraat 10, 5042 SB Tilburg, the Netherlands

**Keywords:** AAA, Acuros XB, Plan quality, Stage I lung cancer, Stage III lung cancer, VMAT

## Abstract

**Introduction:**

The main aim of the current study was to assess the dosimetric accuracy and clinical quality of volumetric modulated arc therapy (VMAT) plans for stereotactic (stage I) and conventional (stage III) lung cancer treatments planned with Eclipse version 10.0 Anisotropic Analytical Algorithm (AAA) and Acuros XB (AXB) algorithm.

**Methods:**

The dosimetric impact of using AAA instead of AXB, and grid size 2.5 mm instead of 1.0 mm for VMAT treatment plans was evaluated. The clinical plan quality of AXB VMAT was assessed using 45 stage I and 73 stage III patients, and was compared with published results, planned with VMAT and hybrid-VMAT techniques.

**Results:**

The dosimetric impact on near-minimum PTV dose (*D*_98%_) using AAA instead of AXB was large (underdose up to 12.3%) for stage I and very small (underdose up to 0.8%) for stage III lung treatments. There were no significant differences for dose volume histogram (DVH) values between grid sizes. The calculation time was significantly higher for AXB grid size 1.0 than 2.5 mm (*p* < 0.01). The clinical quality of the VMAT plans was at least comparable with clinical qualities given in literature of lung treatment plans with VMAT and hybrid-VMAT techniques. The average mean lung dose (MLD), lung *V*_20Gy_ and *V*_5Gy_ in this study were respectively 3.6 Gy, 4.1% and 15.7% for 45 stage I patients and 12.4 Gy, 19.3% and 46.6% for 73 stage III lung patients. The average contra-lateral lung dose *V*_5Gy-cont_ was 35.6% for stage III patients.

**Conclusions:**

For stereotactic and conventional lung treatments, VMAT calculated with AXB grid size 2.5 mm resulted in accurate dose calculations. No hybrid technique was needed to obtain the dose constraints. AXB is recommended instead of AAA for avoiding serious overestimation of the minimum target doses compared to the actual delivered dose.

## Background

Volumetric modulated arc therapy (VMAT) has been shown to be a powerful technique for irradiation of many treatment sites with obtaining higher dose conformity to the tumor while decreasing intra-fraction movements because of shorter delivery times [1-8]. Reliable and accurate dose delivery can be obtained using VMAT as shown by pre-treatment dosimetric plan validations [9]. VMAT could achieve at least comparable clinical plan qualities and skin dose levels than intensity modulated radiotherapy (IMRT) [10-12] and can successfully be used for stereotactic body radiotherapy (SBRT) for patients with stage I Non-Small-Cell Lung Cancer (NSCLC) [2,3,5].

In case of stage III large tumor lung cancers, it is difficult to limit doses to organs at risks (OARs) such as heart and lung. High doses are preferred since local control increased significantly (*p* = 0.02) when patients are treated with higher doses than 64 Gy [13]. Rengan et al. [13] stated that the median survival time for patients treated to 64 Gy or higher was 20 months versus 15 months for those treated to less than 64 Gy. Advanced planning techniques, IMRT and VMAT, have been shown to be able to increase the therapeutic dose with equal toxicity profiles compared to three-dimensional conformal radiotherapy (3DCFRT) [14]. Unfortunately, it is not always possible to deliver doses higher than 60 Gy to the planning target volume (PTV) using 3DCFRT, IMRT and VMAT because of dose limiting organs [14]. De Bree-Balk et al. [14] stated that possibly further improvements could be made by using hybrid techniques which combine two static fields with IMRT or VMAT, as also investigated by Verbakel et al. [15], who made a comparison between conventional static field plans, IMRT, hybrid-IMRT, VMAT and hybrid-VMAT; and by Chan et al. [16], who compared between 3DCFRT, VMAT and hybrid-VMAT. In both studies the VMAT plans consisted of at least 2 arcs and the hybrid plans of a combination of two static fields and IMRT or VMAT. They have concluded that hybrid techniques are superior in dosimetric outcomes for treating stage III lung tumours compared to the other techniques. The treatment planning for these studies has been performed using Varian Eclipse treatment planning version 8 or 10 with Anisotropic Analytical Algorithm (AAA).

We have recently introduced the Varian Eclipse software version 10, with the AAA as well as the Acuros XB (AXB) algorithm for photon dose calculations in our institute. AXB solves the linear Boltzmann transport equation e.g., [17]. The dosimetric accuracy of AXB has already been investigated in several studies [18-22]. Fogliata et al. [19,21] have concluded that AXB gives acceptable characteristics in homogeneous media for small and large fields (range 0.8×0.8 to 40.0×40.0 cm^2^) using comparisons of AXB with AAA and measurements. In heterogeneous situations, the AXB algorithm has been shown to provide a valid and accurate alternative to Monte Carlo calculations for field sizes ranging from 2.5×2.5 to 30.0×30.0 cm^2^[18,20,22].

Immediately after clinical introduction of the Varian Eclipse software, we also clinically introduced VMAT for lung SBRT stage I NSCLC as well as for lung stage III treatments. For this clinical introduction, we investigated the dosimetric accuracy and quality of stereotactic and conventional VMAT planning in Eclipse using AXB and AAA. Routinely, for all our patients, we perform pre-treatment verification measurements using an ionization chamber in the isocentre, combined with film measurements in the isocentre plane.

It was already shown by Gete et al. [23] that AAA calculations can slightly overestimate the minimum PTV dose relative to Monte Carlo calculations with BEAMnrc/DOSXYZnrc for stage I lung tumors (PTV range between 19 to 62 cm^3^) with forward planning with multiple static non-coplanar conformal fields. It has also been shown by VMAT comparison studies that AXB leads to a slightly more accurate dose distribution than AAA [24,25]. For stage III lung tumors (average PTV 690 cm^3^), Fogliata et al. [26] have illustrated that AAA leads to a monitor unit underestimation of approximately 1-2% relative to AXB grid size 2.5 mm using a treatment planning comparison between 3DCFRT, IMRT and VMAT.

Consequently, this suggests that AAA could overestimate the minimum target dose, which leads to lower target coverage than the prescribed dose, in case AXB represents the real dose distribution. Kan et al. [27] have illustrated that AXB was more accurate in predicting secondary build-up near and beyond air/tissue interfaces than AAA, using a comparison with measurements. Bush et al. [22] have shown that AXB was capable of modelling radiotherapy dose deposition in the low density regions. Dose distributions calculated by AXB were in good agreement with BEAMnrc/DOSXYZnrc Monte Carlo dose calculations.

The purpose of this study was threefold. First, we wanted to justify the assumption that AXB represents better the real dose distribution than AAA by cross-checking the finding of Bush et al. [22] with measurements, for calculation grid sizes of 1.0 mm and 2.5 mm. Second, we investigated the dosimetric impact of using AAA instead of AXB, and grid size 2.5 mm instead of 1.0 mm for VMAT treatment plans for stage I and stage III lung tumors. Third, we investigated whether using AXB calculations in Eclipse version 10.0, VMAT results in improved treatment plans compared to VMAT and hybrid-VMAT plans published in literature, again for stage I (lung stereotactic) and stage III (conventional lung) treatments.

## Methods

### Dose calculations

All calculations were performed using the treatment planning system (TPS) Eclipse version 10 with beam algorithms AAA and AXB (Varian Medical Systems, Palo Alto, CA), which was installed on a standard clinical workstation (Dell T5500) with dual 2.40 GHz quad-core Intel processors (E5620), 24 GB RAM, and a 64 bit Windows 7 operating system, in a distributed calculation framework (DCF) network of 3 workstations. The dose reporting mode dose-to-medium *D*_m_ was selected for AXB.

### Justification of the assumption that AXB represents the real dose distribution

Before performing the main parts of our study, we validated the findings of Bush et al. [22] that AXB represents the actual dose delivery by performing percentage depth dose (PDD) measurements using EBT2 film (ISP, Wayne, NJ) in a simple heterogeneous interface phantom, which consisted of three layers: upper and lower of polystyrene with a density of *ρ* = 1.05 g cm^-3^ and thickness of 5 and 7 cm, respectively, and a middle layer of foam, with a low-density *ρ* = 0.03 g cm^-3^ and a thickness of 8 cm. This very low density was chosen to investigate the accuracy of both algorithms in a very extreme situation analogue to Bush et al. [22]. The measurements and Eclipse dose calculations were performed for a field size of 1.0×1.0 cm^2^ and 4.0×4.0 cm^2^ using a 6 MV photon beam (Clinac 2100iX equipped with a Millenium 120-MLC, Varian Medical Systems, Palo Alto, CA) of 200 monitor units and a source to skin distance (SSD) of 100 cm.

### Patient selection

Eight stage I and seven stage III lung patients were selected to investigate the dosimetric impact of using AAA instead of AXB, and grid size 2.5 mm instead of 1.0 mm, for dose calculations of VMAT treatment plans. The average PTV volume was 24.0 cm^3^ (range 5.1 to 56.9 cm^3^) for stage I and 418.9 cm ^3^ (range 140.3 to 762.6 cm^3^) for stage III tumors. OARs were delineated including heart and contra-lateral lung. The clinical quality of the VMAT plans was assessed using 45 stage I lung patients (average PTV 35.4 cm^3^; range 5.5 to 175.4 cm^3^ ) and 73 stage III lung patients (average PTV 344.4 cm^3^; range 25.1 to 1069.0 cm^3^).

### Treatment planning

For the algorithm comparison, optimal VMAT plans were prepared using AAA algorithm grid size 2.5 mm consisting of two 180 degrees arcs using the VMAT optimization tool. Dose prescription was 54 Gy in 5 fractions to the 80% isodose, which covered at least 99% of the PTV, for stage I, and for stage III 66 Gy in 33 fractions, where at least 99% of the PTV received 90% of the prescribed dose. For stage III, an additional constraint of *D*_*≤*1%_ = 72.6 Gy (110% of prescribed dose) was used and the dose to OARs, such as lung and heart, was optimized. The plans were recalculated using the same beam settings and monitor units as the AAA grid size 2.5 mm treatment plans for AAA grid size 1.0 mm, AXB grid size 2.5 mm and AXB grid size 1.0 mm.

In the VMAT optimization process, a Clinical Protocol was used (and if necessary, optimized interactively) with Optimization Objectives for the PTV, heart, lungs and spinal cord (with a margin of 5 mm). Most dose volume histogram (DVH) objectives had a priority of 50, whereas the minimum and maximum PTV dose, defined as *D*_100%_ and *D*_0%,_ had a priority of 250 and the maximum spinal cord dose (10 Gy for stage I and 44 Gy for stage III) had a priority of 150 or 200. For stage III VMAT plans, a MLD objective of 12 Gy with a priority of 200 was applied; and a contra-lateral lung dose *V*_5Gy-cont_ objective of 30% or 25% was used with a priority of respectively 450 or 500 for the 73 stage III patients. This study used the volume of both lungs minus internal target volume (ITV) for the calculation of MLD, and volume of both lungs minus PTV for *V*_20Gy_, and *V*_5Gy_. Most of the time, we applied a “Normal Tissue Objective” with a fall-off of 4 for stage I stereotactic treatments and of 1 for stage III conventional lung treatments. A help volume with a margin of 5 mm around the PTV was introduced with also relatively high priorities when OARs were not spared sufficiently. It was almost never necessary to include constraints for the other OARs.

### Evaluation tools

Dose volume histograms were produced for all plans in order to analyze the doses to the PTV and OARs. The statistical differences were tested for the VMAT treatment plans between AAA grid size 2.5 mm, AAA grid size 1.0 mm, AXB grid size 2.5 mm and AXB grid size 1.0 mm. The statistical significance of the differences was tested with a paired two-tailed student *t*-test with significant level *p* < 0.05.

## Results and discussion

### Justification of the assumption that AXB represents the real dose distribution

**Figure 1:**
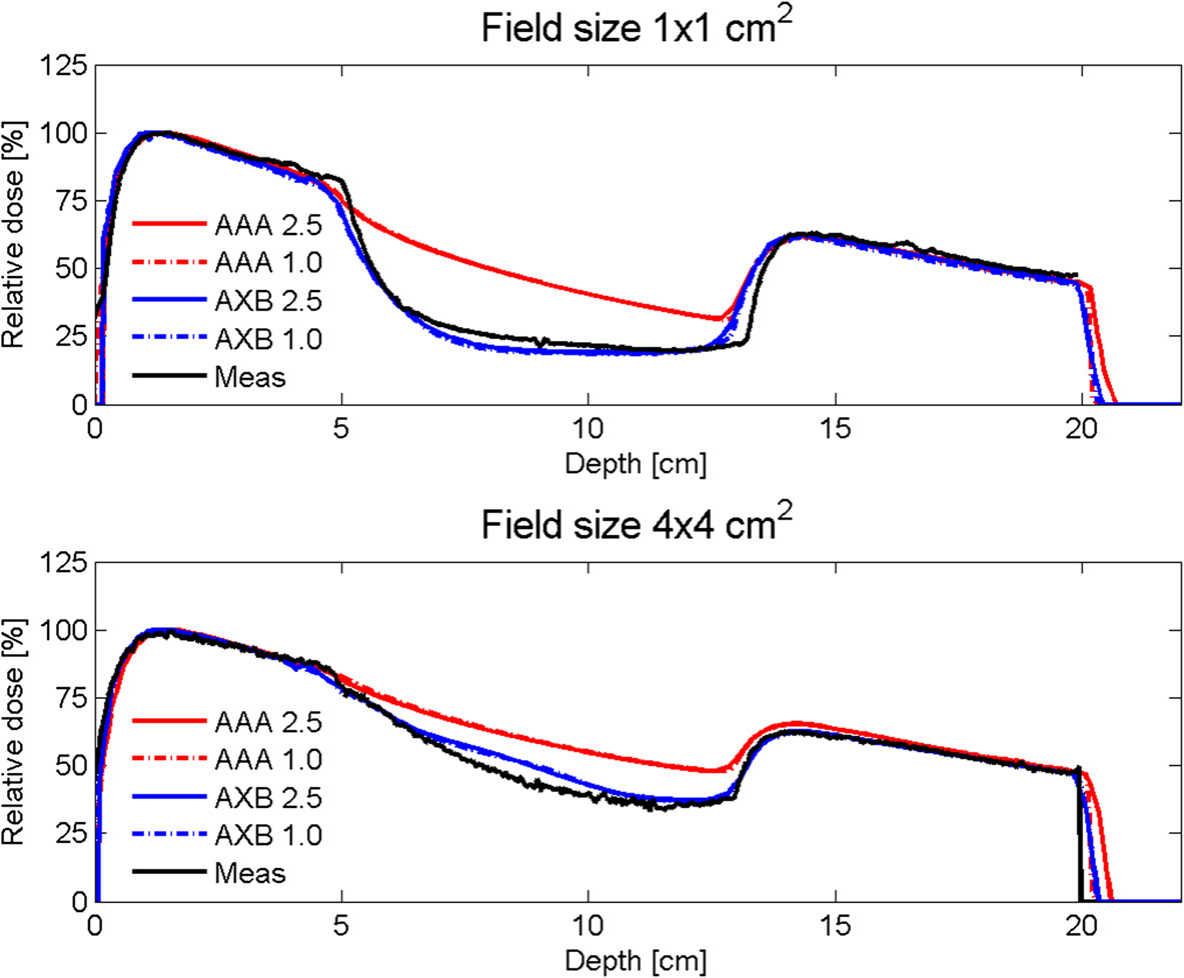
**Measured and calculated percentage depth dose curves in a slab-phantom.** Predicted percentage depth dose curves by AAA and Acuros XB grid sizes 2.5 and 1.0 mm compared to measured data using a slab-phantom with (top) 1.0x1.0 cm^2^ and (bottom) 4.0x4.0 cm^2^ 6 MV AP photon beams.

The analyses in this study to cross-check the findings by Bush et al. [22] confirmed that AXB is much more accurate in heterogeneous situations than AAA (Figure 1). For single fields, there were no large differences between grid size 2.5 mm and grid size 1.0 mm. The relative doses were much higher for AAA than AXB in the middle low density layer (*ρ* = 0.03 g cm^-3^) of the interface phantom. The difference in relative doses were even larger than 20% for a field size of 1.0×1.0 cm^2^ and larger than 10% for a field size of 4.0×4.0 cm^2^. Bush et al. [22] have also published large relative dose differences, e.g. larger than 30% for a very low density media (*ρ* = 0.001 g cm^-3^) and a field size of 4.0×4.0 cm^2^.

Therefore, it is now shown by simulations and measurements that calculations using AXB represent better the actual delivered dose distribution in case of narrow beams and heterogeneous situations than AAA. Of course, in most patients real lung density will be larger than the low density taken in this study (*ρ* = 0.03 g cm^-3^), and differences between AXB and AAA will be smaller as also stated by Bush et al. [22].

### Dosimetric impact of algorithms and grid sizes on clinical lung VMAT plans

Dose volume histograms were produced for all plans so that the relative differences between algorithms (AAA and AXB) and grid sizes (2.5 and 1.0 mm) could be analyzed. The dose calculation time by AAA grid size 1.0 mm in comparison to AAA grid size 2.5 mm, AXB grid size 2.5 mm and AXB grid size 1.0 was much larger. For example for stage I, the calculation times were 7, 10 and 3 times larger with AAA grid size 1.0 mm in comparison to AAA grid size 2.5 mm, AXB grid size 2.5 mm and AXB grid size 1.0 mm. We have decided to focus on the dose distribution differences between AAA grid size 2.5 mm and AXB grid size 2.5 mm; and on the dose distribution differences between AXB grid size 2.5 mm and AXB grid size 1.0 mm since AAA grid size 1.0 mm will probably never be used clinically and the differences with AAA grid size 2.5 mm were very small.

**Figure 2:**
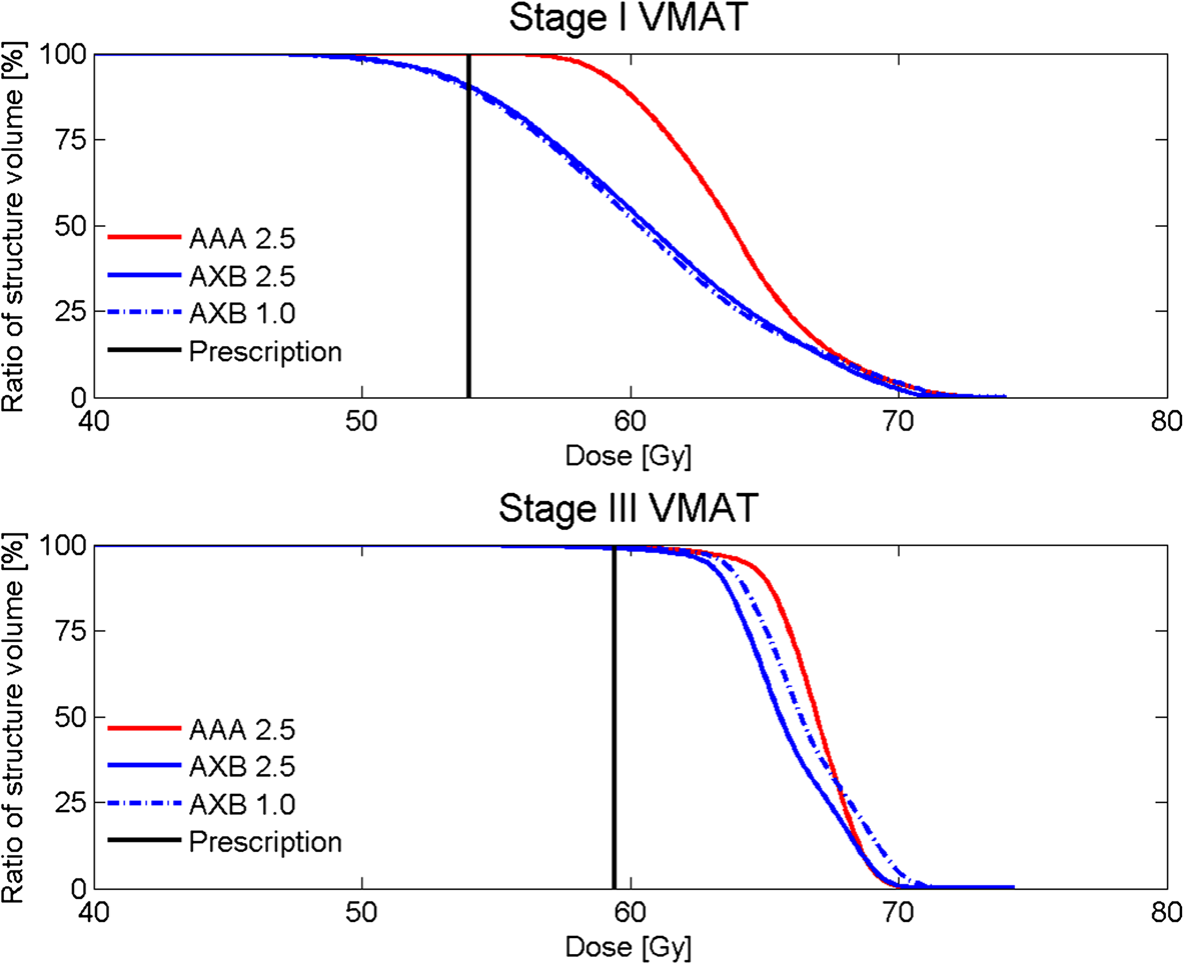
**Dose volume histogram comparisons of PTV doses between algorithms and grid sizes.** Comparison of dose volume histograms of the planning target volume (PTV) between AAA grid size 2.5 mm, Acuros XB grid size 2.5 mm and Acuros XB grid size 1.0 mm of (top) a stage I and (bottom) a stage III lung patient. The black vertical lines indicate the prescription: The dose that has to cover 99% of the PTV, respectively, 54.0 Gy for stage I and 59.4 Gy for stage III.

A summary of the relative dose differences between algorithms and grid sizes is given in Table 1. For individual stage I lung patients differences occurred in dose distributions between AXB and AAA. The maximum difference of near-minimum PTV dose (*D*_98%_) between AXB grid size 2.5 mm and AAA grid size 2.5 mm was −7.1 Gy corresponding to a relative difference of −12.3%, which indicated a serious underdosage of the delivered dose when this patient would have been planned with the AAA algorithm (Table 1, Figure 2). This finding is similar to results published by Gete et al. [23]. They observed a 12.8% lower minimum PTV dose using Monte Carlo simulations than using AAA version 8.6 calculations for one patient plan. These relative differences can be explained by the significantly improved accuracy of AXB under the conditions of electronic disequilibrium compared to AAA. AAA only predicts little secondary build-up at regions beyond low-density media like lung [22,26,27].

**Table 1:** Relative differences between algorithms and grid sizes for lung cancer patients

	**Relative difference**	***p*-value**	**Relative difference**	***p*-value**
	**(AXB2.5 -AAA2.5)/ AAA2.5×100%**		**(AXB1.0 - AXB2.5)/ AXB2.5×100%**	
	**Average ± SDV (min-max)**		**Average ± SDV (min-max)**	
**Stage I (N = 8)**				
PTV				
*D*_98%_	−3.2% ± 4.0% (−12.3% – 0.5%)	0.06	0.7% ± 1.3% (−0.8% – 3.1%)	0.19
*D*_2%_	0.2% ± 1.2% (−2.1% – 1.3%)	0.69	0.9% ± 0.4% (0.3% – 1.4%)	<0.01^*^
*D*_mean_	−0.6% ± 2.2% (−4.9% – 1.6%)	0.46	0.2% ± 0.5% (−0.6% – 1.2%)	0.45
Total lung				
*V*_5Gy_	1.3% ± 1.9% (−1.9% –3.2%)	0.12	0.1% ± 0.6% (−0.8% – 1.1%)	0.73
*V*_20Gy_	2.0% ± 2.3% (0.0% – 5.7%)	0.05^*^	−0.7% ± 1.2% (−2.7% – 0.0%)	0.17
MLD	0.0% ± 0.5% (−0.7% – 0.7%)	0.60	0.1% ± 0.2% (0.0% – 0.4%)	0.08
Time				
Calculation	−39.2% ± 13.5% (−50.3% – –8.2%)	<0.01^*^	304.7% ± 48.7% (226.9% – 345.8%)	<0.01^*^
**Stage III (N = 7)**				
PTV				
*D*_98%_	−0.3% ± 0.7% (−0.8% – 1.3%)	0.33	0.2% ± 0.8% (−1.4% – 0.9%)	0.57
*D*_2%_	−0.6% ± 2.0% (−2.1% – 3.0%)	0.47	0.6% ± 0.4% (−0.1% – 1.0%)	<0.01^*^
*D*_mean_	−0.8% ± 0.7% (−1.6% – 0.3%)	0.02^*^	0.1% ± 0.3% (−0.5% – 0.4%)	0.40
Total lung				
*V*_5Gy_	−1.8% ± 1.7% (−4.8% – 0.3%)	0.03^*^	−0.4% ± 0.7% (−1.5% – 0.4%)	0.16
*V*_20Gy_	0.2% ± 0.7% (−0.5% – 1.5%)	0.69	0.0% ± 0.0% (0.0% –0.0%)	1.00
MLD	−0.5% ± 0.6% (−1.3% – 0.0%)	0.09	0.2% ± 0.3% (0.0% – 0.6% )	0.17
Time				
Calculation	−31.4% ± 9.9% (−44.4% – –17.9%)	<0.01^*^	556.9% ± 91.1% (412.3% – 646.0%)	<0.01^*^

^*^Significant relative difference tested by two-tailed student *t*-test.

AXB showed a slightly but significant (*p* < 0.05) higher *V*_20Gy_ (volume of both lungs minus PTV with a dose of 20 Gy or more) value than AAA for stage I patients. As a result, AAA grid size 2.5 mm treatment plans might be clinically approved with the real *V*_20Gy_ being slightly too high. The differences in lung doses calculated by AAA and AXB were patient dependent since they were dependent on field size, location of the target inside the lung and the density of the lung. Depending on the actual combination of field size, target location and lung density, AAA can over- or underestimate the lung dose, as also stated by Bush et al. [22]. The relative dose differences between both models for the other OARs were small. However, there was a large difference in calculation time between both algorithms. The dose calculation times required by AAA grid size 2.5 mm were about 2 times the calculation times required by AXB grid size 2.5 mm.

In case of stage III patients, the difference in PTV doses were smaller than in case of stage I patients (Table 1, Figure 1) due to, e.g. larger fields and tumor sizes, resulting in smaller errors in the AAA algorithm. The differences in OAR doses were also small. There was again a significant (*p* < 0.01) difference in calculation time between AAA and AXB. The average final dose calculation time was respectively, 6 and 4 minutes, for AAA grid size 2.5 mm and AXB grid size 2.5 mm.

Only small significant differences were found between AXB grid size 2.5 mm and AXB grid size 1.0 mm for near-maximum PTV dose (*D*_2%_) (Table 1, Figure 2), while the calculation time will increase drastically when grid size 1.0 mm was used instead of grid size 2.5 mm. The average calculation time was 2 and 8 minutes for stage I patients with grid size 2.5 mm and 1.0 mm, respectively, and 4 and 27 minutes for stage III patients with grid size 2.5 mm and 1.0 mm.

Therefore, we recommend using AXB grid size 2.5 mm for VMAT planning of lung tumors, since this results in accurate dose values with acceptable calculation times.

### Clinical quality of VMAT

The dose volume quantities of 45 stage I and 73 stage III lung patients planned with VMAT AXB grid size 2.5 mm were analyzed. The average MLD, total lung *V*_20Gy_ and total lung *V*_5Gy_ were 3.6 Gy, 4.1% and 15.7% for stage I and 12.4 Gy, 19.3% and 46.6% for stage III. The prescribed dose of 66 Gy could be delivered to all patients. The MLD was between 16.0 and 18.5 Gy for 15% of the stage III patients, for all other patients, the MLD was lower than 16 Gy.

It was difficult to compare the obtained plan qualities with literature since different patients were used. However, when we compared the dose volume quantities with published studies about VMAT techniques for stage I and stage III NSCLC patients [2,3,16,28], we concluded that the plan qualities were at least comparable. For example, McGrath et al. [3] have published an average MLD for stage I tumors of 4.6 Gy (with dose prescription: 99% of the PTV_ITV_ has to receive more than 43.2 Gy) with VMAT. In our study the average MLD was 3.6 Gy (with a higher dose description: 99% of the PTV has to receive more than 54.0 Gy).

For stage III patients, the clinical quality of treatment plans is a trade-off between high doses to gross tumour volume and limiting treatment related pneumonitis (TRP). Doses higher than 64 Gy are preferred [13] which could be obtained for all 73 clinical patients in this study. Simultaneously, lung doses should be minimized since these influenced the post-radiation acute TRP. Several studies describe analyses to determine indicators for predicting TRP [29-33]. Different predictors were indicated in these studies, e.g. *V*_5Gy-cont_ by Song et al. [29], *V*_5Gy_ by Wang et al. [30], MLD and *V*_30Gy_ by Kim et al. [31], and *V*_10Gy_ by Shi et al. [32] and Spych et al. [33]. Song et al. [29] have shown with an extensive multivariate analysis including *V*_5Gy_, *V*_10Gy_, *V*_13Gy_, *V*_15Gy_, V_20Gy_ and MLD for total lung, ipsilateral lung and contra-lateral lung that *V*_5Gy-cont_ was the only remaining significant factor associated with TRP. They concluded that *V*_5Gy-cont_ should be kept as low as possible and they suggested a cut-off value of 60% since incidences of grade ≥ 3 pneumonitis were 35% and 0%, respectively, for *V*_5Gy-cont_ ≤ 60% and *V*_5Gy-cont_ > 60% (*p* = 0.01).

In this study, a *V*_5Gy-cont_ planning objective of 30% or 25% was used with priority of 450 or 500 for the 73 stage III patients planned with VMAT. Adding this objective decreased significantly the MLD, *V*_5Gy_ and *V*_5Gy-cont_ (*p* < 0.01) from 12.7 Gy, 53.5% and 47.7% to 12.4 Gy, 46.6% and 35.6%, respectively. The *V*_5Gy-cont_ was larger than 60% for only one patient. Kim et al. [31] have used a MLD cut-off value of 16 Gy and indicated that the actual incidence of lung toxicity of grade ≥ 2 was 8% for MLD ≤ 16 Gy and 54% for MLD > 16 Gy (*p* < 0.01). Wang et al. [30] have determined an incidence of grade ≥ 3 TRP at 1 year of 13% for MLD ≤ 16.5 Gy and of 36% for MLD >16.5 Gy (*p* = 0.02). We have obtained a MLD ≤ 16.5 Gy for 90.4% of the patients and the maximum MLD was 18.3 Gy.

The lung dose values, MLD, *V*_20Gy_, *V*_5Gy_ and *V*_5Gy-cont_ were compared to lung dose values published in the literature using VMAT and hybrid-VMAT techniques. It is shown in Table 2 that the clinical VMAT plans of this study were at least comparable to the plans obtained with VMAT and hybrid-VMAT discussed in literature [14-16]. Historically, all our stage III plans were normalised as: 99% of the PTV is covered by 90% of the prescribed dose (66 Gy). Our normalisation is in close agreement with the recommended normalisation method by ICRU 83 [34], to the mean PTV dose. For our stage III patients, the mean PTV dose was 101.2% ± 1.4%. This implies that our normal tissue DVH values will even be slightly better when we use ICRU 83 for normalisation in the future.

**Table 2:** Comparison of stage III VMAT and hybrid-VMAT plans

**Study**	**Method**	**N**	***D***_**prescibed **_**[Gy]**	***V***_**PTV **_**[cm**^**3**^**]**	**MLD [Gy]**	***V***_**20Gy **_**[%]**	***V***_**5Gy **_**[%]**	***V***_**5Gy-cont **_**[%]**
De Bree-Balk et al. [14]^1^	VMAT	20	66	838^5^	20.0	36.6	NA	69.6
Verbakel et al. [15]^2^	VMAT	14	66	779	NA	30.3 ± 5.7	NA	44.6 ± 9.0
	H-VMAT	14	66	779	NA	30.1 ± 5.8	NA	36.2 ± 15.0
Chan et al. [16]^3^	VMAT	24	60	508	14.4 ± 2.9	25.4 ± 6.0	64.0 ± 15.4	NA
	H-VMAT	24	60	508	14.0 ± 2.9	23.3 ± 5.3	59.5 ± 16.7	NA
This study^4^	VMAT	73	66	344	12.4 ± 3.5	19.3 ± 6.8	46.6 ± 10.6	35.6 ± 7.1
This study^4^*V*_PTV_ > 500 cm^3^	VMAT	13	66	678	14.5 ± 2.1	22.1 ± 6.4	52.1 ± 10.3	38.7 ± 10.0

Average values and standard deviations are given.

^1^[14] Use the volume of both lungs minus CTV for MLD and *V*_20Gy_; PTV *V*_95%_ > 99% and PTV *V*_107%_ < 1%.

^2^[15] Use the volume of both lungs minus PTV for *V*_20Gy_; PTV *V*_95%_ > 97% and PTV *V*_107%_ < 5%.

^3^[16] Use the volume of both lungs minus PTV for MLD, *V*_20Gy_ and *V*_5Gy_; *D*_98%_ > 57.0 Gy and *D*_2%_ < 64.2 Gy.

^4^ This study uses the volume of both lungs minus ITV for MLD, and volume of both lungs minus PTV for *V*_20Gy_ and *V*_5Gy_.

PTV *V*_90%_ > 99% and PTV *V*_110%_ ≤ 1%.

^5^ Median instead of average value is given.

This study illustrates that it is possible with two 180 degree arcs to obtain clinical plan qualities compared to VMAT and hybrid-VMAT plans described in literature. Generating acceptable plans using VMAT with two arcs of 180 degrees required only 1 hour for plan optimization and dose calculations. Consequently, our clinical VMAT plans show comparable clinical plan quality as hybrid VMAT techniques, therefore being a quick and easy alternative.

## Conclusions

We investigated the quality of VMAT treatment plans using Eclipse treatment planning system version 10.0 for stage I and III lung patients. All plans consisted of 2 partial arcs of 180 degrees. We showed that the AXB calculation algorithm was preferable to AAA since possible PTV underdosage, as a result of inaccurate AAA calculations, can be avoided. In addition, the calculation time was much shorter for AXB. For clinical VMAT lung plans, the quality and accuracy of AXB grid size 2.5 mm was comparable with AXB grid size 1.0 mm. However, the calculation time increases drastically when grid size 1.0 mm was used.

The VMAT plans were compared to published treatment planning studies. The clinical VMAT AXB grid size 2.5 mm plans obtained in this study were at least comparable to the published planning studies, e.g. planned with hybrid-VMAT, therefore being a quick and easy alternative for this technique.

## Competing interest

The authors declare no conflict of interest.

## Authors’ contributions

PK designed the set-up of the study, performed the data collection and analyses, carried out the comparisons and drafted the manuscript. SH generated most of the treatment plans. ME supervised the project, helped to draft the manuscript, participated in the design of the study, data collection, treatment planning and analyses. All authors read and approved the final manuscript.
